# Characterizing Mechanisms of Ischemia in Patients With Myocardial Bridges

**DOI:** 10.1161/CIRCINTERVENTIONS.123.013657

**Published:** 2023-11-06

**Authors:** Aish Sinha, Haseeb Rahman, Ronak Rajani, Ozan M. Demir, Matthew Li KamWa, Holly Morgan, Saad M. Ezad, Howard Ellis, Dexter Hogan, Ankur Gulati, Ajay M. Shah, Amedeo Chiribiri, Andrew J. Webb, Michael Marber, Divaka Perera

**Affiliations:** British Heart Foundation Centre of Excellence, School of Cardiovascular Medicine and Sciences, King’s College London, United Kingdom (A.S., H.R., O.M.D., M.L.K., H.M., S.M.E., H.E., A.M.S., A.C., A.J.W., M.M., D.P.).; Guy’s and St. Thomas’ NHS Foundation Trust, London, United Kingdom (R.R., D.H., A.G., D.P.).

**Keywords:** angina, endothelium, microcirculation, myocardial bridging

## Abstract

**BACKGROUND:**

Myocardial bridges (MBs) are prevalent and can be associated with acute and chronic ischemic syndromes. We sought to determine the substrates for ischemia in patients with angina with nonobstructive coronary arteries and a MB in the left anterior descending artery.

**METHODS:**

Patients with angina with nonobstructive coronary arteries underwent the acquisition of intracoronary pressure and flow during rest, supine bicycle exercise, and adenosine infusion. Coronary wave intensity analysis was performed, with perfusion efficiency defined as accelerating wave energy/total wave energy (%). Epicardial endothelial dysfunction was defined as a reduction in epicardial vessel diameter ≥20% in response to intracoronary acetylcholine infusion. Patients with angina with nonobstructive coronary arteries and a MB were compared with 2 angina with nonobstructive coronary arteries groups with no MB: 1 with coronary microvascular disease (CMD: coronary flow reserve, <2.5) and 1 with normal coronary flow reserve (reference: coronary flow reserve, ≥2.5).

**RESULTS:**

Ninety-two patients were enrolled in the study (30 MB, 33 CMD, and 29 reference). Fractional flow reserve in these 3 groups was 0.86±0.05, 0.92±0.04, and 0.94±0.05; coronary flow reserve was 2.5±0.5, 2.0±0.3, and 3.2±0.6. Perfusion efficiency increased numerically during exercise in the reference group (65±9%–69±13%; *P*=0.063) but decreased in the CMD (68±10%–50±10%; *P*<0.001) and MB (66±9%–55±9%; *P*<0.001) groups. The reduction in perfusion efficiency had distinct causes: in CMD, this was driven by microcirculation-derived energy in early diastole, whereas in MB, this was driven by diminished accelerating wave energy, due to the upstream bridge, in early systole. Epicardial endothelial dysfunction was more common in the MB group (54% versus 29% reference and 38% CMD). Overall, 93% of patients with a MB had an identifiable ischemic substrate.

**CONCLUSIONS:**

MBs led to impaired coronary perfusion efficiency during exercise, which was due to diminished accelerating wave energy in early systole compared with the reference group. Additionally, there was a high prevalence of endothelial and microvascular dysfunction. These ischemic mechanisms may represent distinct treatment targets.

**WHAT IS KNOWN**

- Myocardial bridges can be associated with microvascular dysfunction, coronary artery spasm, and proximal vessel atherosclerosis.
- The hemodynamic effects of the tunneled segment during exercise are not known.

**WHAT THE STUDY ADDS**

- Patients with myocardial bridges demonstrate impaired coronary perfusion efficiency during exercise.
- The tunneled segment leads to perturbation of accelerating wave energy in early systole during exercise, which is the main determinant of impaired perfusion efficiency.
- Endothelial dysfunction is highly prevalent in patients with myocardial bridges, with the myocardial bridge muscle index being independently associated with it.

Angina with nonobstructive coronary arteries (ANOCA) is a common clinical problem and comprises several distinct pathophysiological entities, including coronary microvascular disease (CMD), coronary artery spasm, and myocardial bridging. Myocardial bridging due to intramyocardial passage of varying lengths of an epicardial artery is a common anatomic variant, found in up to 30% of patients on coronary computed tomography angiography (CCTA) imaging,^1^ predominantly in the left anterior descending (LAD) artery at the mid vessel. The intramyocardial segment of the vessel is known as the tunneled segment. Historically, myocardial bridges (MBs) have been considered a benign entity as myocardial perfusion predominantly occurs during diastole and MBs are thought to only alter vessel caliber during systole. However, growing evidence suggests that MBs are not always benign and have been associated with chronic intermittent angina, as well as acute ischemic presentations.^2^ Several mechanisms have been postulated,^2^ including delayed decompression of the tunneled segment in diastole leading to luminal narrowing akin to obstructive coronary artery disease,^3^ predisposition to coronary artery spasm,^4^ increased propensity to atherosclerotic coronary artery disease proximal to the bridged segment due to perturbed wall shear stress,^4^ and the venturi effect leading to reduced septal blood flow.^5^ However, while a handful of studies have assessed the physiological response to adenosine and dobutamine stress, using pressure as a surrogate of flow, coronary flow during exercise has not been specifically and systematically evaluated in patients with MBs. Our study aimed to characterize the mechanisms that lead to myocardial ischemia in patients with ANOCA and a MB (MB group) during physical exercise using wave intensity analysis to describe patterns of cardiac-coronary coupling. We compared these findings to those in patients with ANOCA but no MB, in turn classified as patients with CMD (CMD group: coronary flow reserve [CFR], <2.5) or normal CFR (reference group: CFR, ≥2.5), respectively. Better understanding of the mechanisms causing ischemia may allow the development of stratified therapies for this underserved patient population.

## METHODS

### Study Population

The data that support the findings of this study are available from the corresponding author upon reasonable request. We enrolled consecutive patients presenting with typical angina and a MB in the LAD artery, identified on prior CCTA imaging or invasive coronary angiography between October 2021 and September 2023 (MB group). Additionally, we enrolled consecutive patients presenting with typical angina without a MB between December 2013 and July 2018 (CMD and reference groups). Inclusion criteria were angina, preserved left ventricular ejection fraction (>50%), and nonobstructive coronary arteries (fractional flow reserve, >0.80). Exclusion criteria were chronic kidney disease (estimated glomerular filtration rate, <30 mL/min per m^2^), significant valvular disease, history of acute coronary syndrome, previous revascularization, and cardiomyopathy. All patients provided written informed consent in accordance with the protocols approved by the UK National Research Ethics Service (20/LO/1294 and 17/LO/0203).

### Intracoronary Physiology Assessment

**Figure 1.**
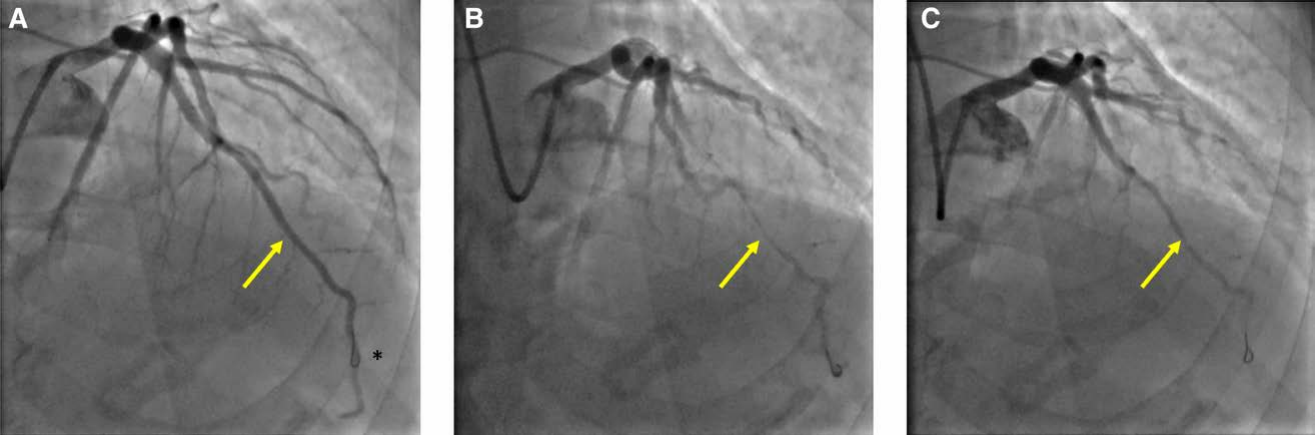
**Example of a myocardial bridge during coronary angiography. A**, Complete decompression of the tunneled segment during mid-late diastole. **B**, Complete obliteration of the tunneled segment throughout systole. **C**, Evidence of vasospasm within the tunneled segment (throughout cardiac cycle) with acetylcholine infusion. *Looped Combowire.

Our protocol for systematic evaluation of ANOCA has been described in full previously.^6^ Briefly, all coronary physiology measurements were made in the LAD artery. A 0.014-inch dual sensor-tipped intracoronary guidewire (Combowire, Philips Volcano, CA) was used for the measurement of distal coronary pressure (Pd) and average peak flow velocity (APV). Aortic pressure (Pa) was measured via the fluid-filled guide catheter. All patients received 1 mg intravenous midazolam, 400 to 600 µg intracoronary isosorbide dinitrate, and 70 U/kg unfractionated heparin before angiography and physiology assessment. Coronary hemodynamic measurements were recorded under resting conditions, during intravenous adenosine-mediated hyperemia (140 µg/kg per min) and continuously during bicycle exercise, using a specially adapted supine ergometer (Ergosana, Bitz, Germany) attached to the cardiac catheter laboratory table. The exercise began at a workload of 30 W and increased every 2 minutes by 20 W. Where lower limb muscle fatigue restricted increasing workloads, resistance was fixed at the maximum tolerated level and exercise continued until exhaustion.^7^ We also assessed patients’ coronary endothelial function using graded intracoronary infusions of acetylcholine (18 µg/mL at 1 mL/min for 2 minutes followed by 2 mL/min for 2 minutes). All intracoronary acetylcholine measurements were made at least 15 minutes after the intracoronary nitrate injection. Contrast coronary angiography was performed at each stage; an example of the stages in a patient with MB is shown in Figure 1.

### Analysis of Coronary Physiological Data

Signals were sampled at 200 Hz, with data exported into a custom-made study manager program (Academic Medical Centre, University of Amsterdam, the Netherlands) and analyzed on custom-made software: Cardiac Waves (Kings College London, UK). CFR was derived as hyperemic APV/basal APV, and CMD was defined as CFR <2.5.^6^ Hyperemic (minimal) microvascular resistance was calculated as Pd/APV during hyperemia. The steady-state Pd/Pa ratio during peak exercise was termed exercise Pd/Pa, and exercise flow reserve was calculated as the ratio of volumetric coronary blood flow (CBF) during peak exercise compared with resting CBF. These were used to compare pancyclic pressure losses and CBF, respectively, between the MB, CMD, and reference groups during exercise. As exercise and acetylcholine infusion can lead to both vasodilatation and vasoconstriction of the epicardial arteries, volumetric CBF incorporating vessel diameter was calculated during the assessment of exercise and acetylcholine flow reserve, respectively (cross-sectional area×APV×0.5). Epicardial endothelial function was defined as the percentage change in vessel caliber (continuous variable), and epicardial endothelial dysfunction as a reduction in coronary luminal diameter by ≥20% (binary classification), in response to intracoronary acetylcholine infusion,^4^ 20% being the recognized limit of precision for quantitative coronary angiography. Microvascular endothelial dysfunction was defined by diminished acetylcholine flow reserve, the ratio of volumetric CBF in response to acetylcholine infusion compared with resting CBF, with acetylcholine flow reserve, ≤1.5 representing microvascular endothelial dysfunction.^8,9^

Wave intensity was calculated as the product of the time derivatives of distal coronary pressure (dPd/dt) and flow velocity (dU/dt), as dPd/dt×dU/dt, with wave separation performed as previously described.^7^ For each patient, 5 dominant waves were identified and included in our analysis: (1) backward compression wave, causing flow deceleration due to compression of microvasculature during isovolumetric contraction; (2) forward compression wave, causing flow acceleration due to increased aortic pressure in early systole; (3) forward expansion wave, causing flow deceleration associated with the fall in aortic pressure in late systole; (4) backward expansion wave, causing flow acceleration due to decompression of the microvasculature in early diastole; (5) late forward compression wave, causing flow acceleration due to augmentation of the aortic pressure during aortic valve closure in diastole (Figure S1). Perfusion efficiency was calculated as the percentage of accelerating wave energies in relation to the total wave energies (%), using areas under the respective curves.

### CCTA Imaging

All CCTA scans were acquired by retrospective ECG-gated spiral scan mode using single- and dual-source CT systems. Patients had 400 µg sublingual nitroglycerin and metoprolol administered with a heart rate goal <60 bpm before image acquisition. Reconstructed CCTA images were reevaluated on an external workstation by 2 experienced radiologists. Diastolic data sets were reviewed. Multiplanar and curved-planar reformations were used for the assessment of the MB in 2 planes: 1 parallel and 1 perpendicular to the vessel’s course. MBs were graded according to the criteria proposed by Kim et al^10^: LAD within the interventricular groove and in contact with the myocardium (1, partial coverage), full encasement of LAD but without visibly overlying myocardium (2, unroofed), and full encasement of LAD with visibly overlying myocardium (3, full coverage). The MB location was determined by measuring the distance from the LAD ostium to the bridge’s entrance. The MB length was measured in millimeters along the vessel axis from the disappearance of the epicardial fat plane proximally to its reemergence distally. We calculated the MB muscle index (MMI) as MB length (mm)×MB coverage grade.^4^ An example of a MB on CCTA is demonstrated in Figure S2.

### Statistical Analyses

The sample size was estimated for the primary outcome measure, exercise perfusion efficiency. Assuming a distribution of 1:1, 26 patients in the MB group and 26 patients in the reference group provided 80% power (α=0.05) to detect a minimum difference in change in exercise perfusion efficiency (exercise, resting perfusion efficiency) of 8% (predicted SD, 10%)^7^ between the reference and MB groups. The normality of data was assessed using the Kolmogorov-Smirnov test. Normally distributed continuous data are presented as mean±SD, unless specified otherwise, and compared using the independent sample Student *t* test. Nonnormally distributed data are presented as median (interquartile range) and compared using the Mann-Whitney *U* test (unpaired analyses) or the Wilcoxon matched-pairs signed-rank test (paired analyses). Categorical variables are presented as n (%) and compared using the χ^2^ test. Continuous end points were compared with the 2-sample *t* test of the difference between groups; the findings are reported as the difference in mean change between study groups with 95% CIs and *P* values. Linear regression was performed using univariable and multivariable analysis, with epicardial endothelial function as the continuous end point, and reported as standardized coefficients (95% CI). Analyses were performed using SPSS Statistics 27 (IBM, NY) and GraphPad Prism software version 9.0 (GraphPad Software, San Diego, CA). We deemed *P*<0.05 to be significant.

## RESULTS

### Baseline Characteristics

**Figure 2.**
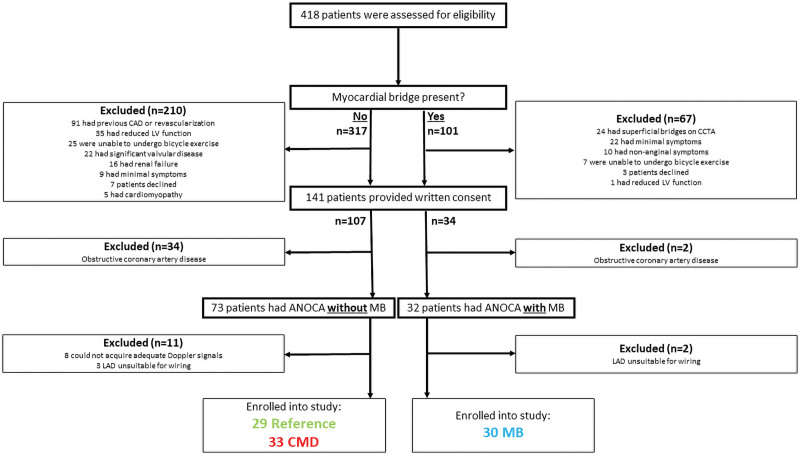
**Study screening and recruitment numbers.** ANOCA indicates angina with nonobstructive coronary arteries; CAD, coronary artery disease; CCTA, coronary computed tomography angiography; CMD, coronary microvascular disease; LAD, left anterior descending; LV, left ventricular; and MB, myocardial bridge.

Of 418 patients screened, 141 were found to meet clinical eligibility criteria. Once these patients underwent invasive physiological assessment, 105 were found to have ANOCA, of which 73 did not have a MB and 32 did have a MB. After excluding patients on account of poor Doppler signals or inability to pass the Combowire into the distal LAD artery, 92 patients were recruited into the study: 30 in the MB group, 33 in the CMD group, and 29 in the reference group (Figure 2). The groups were well matched for age and cardiovascular risk factors. There were more women in the CMD and control groups, compared with the MB group (Table 1). Twelve patients in the MB group underwent a CCTA scan before coronary angiography; in this cohort, the tunneled segment length, depth, and MMI were 29±9, 2±1, and 79±30 mm, respectively.

**Table 1.**
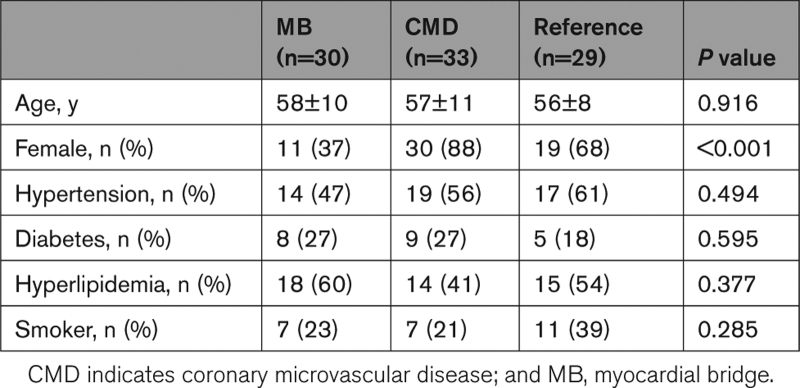
Patient Demographics

### Whole Cardiac Cycle Physiology Measurements

Epicardial physiology measurements confirmed the absence of obstructive coronary disease in all patients (as per eligibility criteria), but the MB group had lower Pd/Pa and fractional flow reserve values than the reference group. CFR in the MB group was 2.5±0.5, which was lower than the reference group (3.2±0.6) but higher than the CMD group (2.0±0.3; *P*<0.001); 14 patients (47%) in the MB group had a CFR <2.5. Minimal microvascular resistance was similar between the MB and reference groups (Table 2).

**Table 2.**
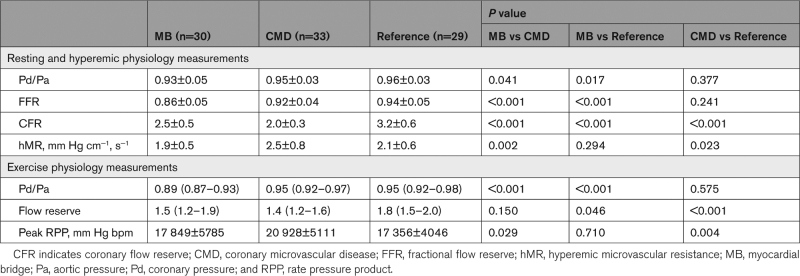
Coronary Physiology Parameters

Patients with a MB had the lowest exercise Pd/Pa (0.89 [0.87–0.93]; *P*<0.001 versus both CMD and reference groups). The MB and CMD groups had similar exercise flow reserve (1.5 [1.2–1.9] versus 1.4 [1.2–1.6]; *P*=0.150), both were lower than the reference group (Table 2).

### Phasic Cycle Physiology Measurements

**Figure 3.**
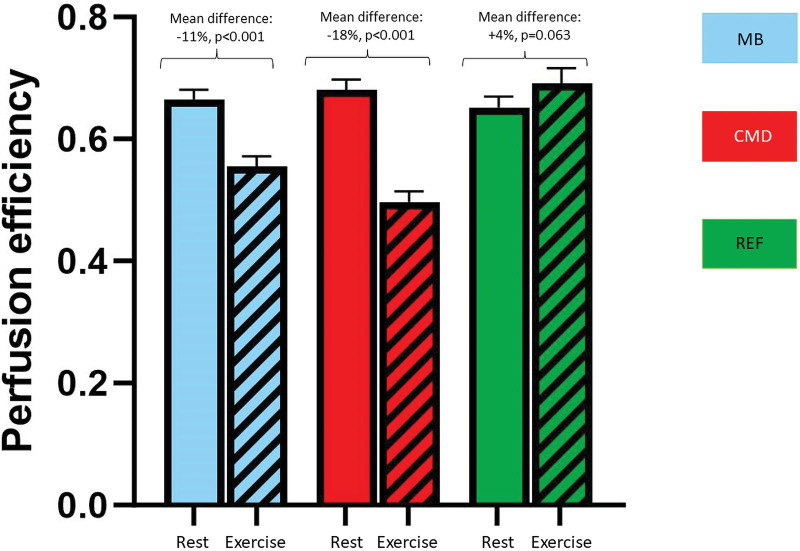
**Coronary perfusion efficiency during exercise in the myocardial bridge (MB), coronary microvascular disease (CMD), and reference groups.** Data are presented as mean±SEM. Colored bar chart key: blue MB group, red CMD group, and green reference group. REF indicates reference.

**Figure 4.**
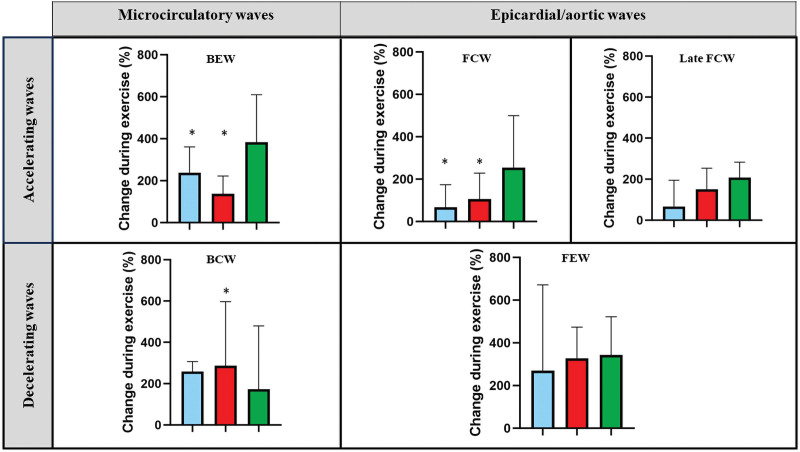
**Changes in coronary wave energies during exercise in the myocardial bridge (MB), coronary microvascular disease (CMD), and reference groups. Data are presented as median (interquartile range).** Colored bar chart key: blue MB group, red CMD group, and green reference group. BCW indicates backward compression wave; BEW, backward expansion wave; FCW, forward compression wave; FEW, forward expansion wave; and late FCW, late forward compression wave. *Significant difference compared with the reference group (*P*<0.05).

**Figure 5.**
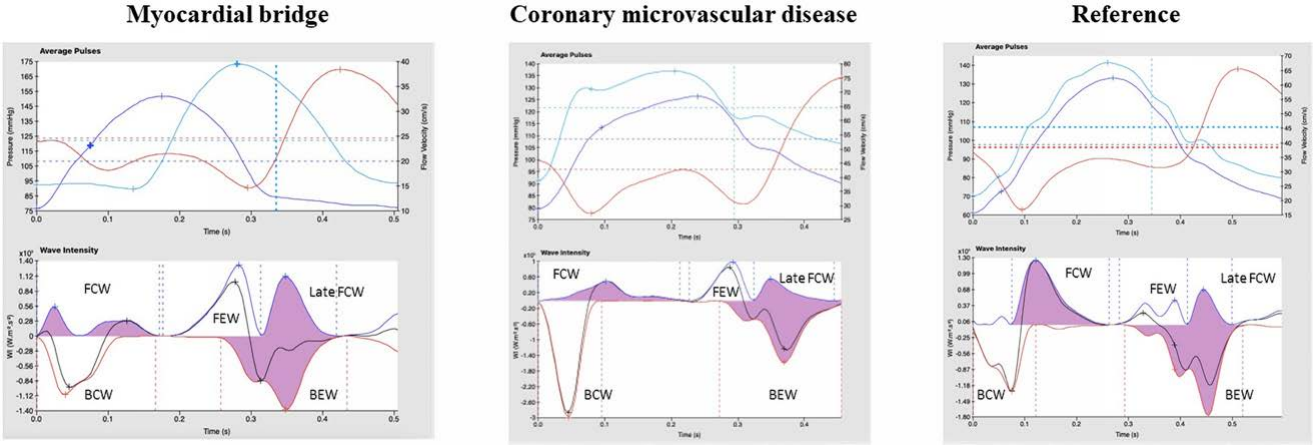
**Comparison of wave intensity analysis, during exercise, between the 3 groups.** Examples of wave intensity analysis during peak exercise in patients with myocardial bridge (MB; left), coronary microvascular disease and no MB (middle), and normal coronary flow reserve and no MB (right). Ensemble averaged aortic pressure (**top**, light blue), distal coronary pressure (**top**, dark blue), flow velocity (**top**, red), and wave intensity analysis (**bottom**). BCW indicates backward compression wave; BEW, backward expansion wave; FCW, forward compression wave; and FEW, forward expansion wave.

By wave intensity analysis, the resting perfusion efficiencies were similar in the MB, CMD, and reference groups (66±9%, 68±10%, and 65±9%; *P*=0.513). In the reference group, perfusion efficiency was numerically enhanced during exercise (from 65±9% to 69±13%; *P*=0.063). In contrast, perfusion efficiency was attenuated during exercise in the MB group (from 66±9% to 55±9%; *P*<0.001) and in the CMD group (from 68±10% to 50±10%; *P*<0.001; Figure 3). The difference in change in perfusion efficiency between the MB and reference groups (ie, delta perfusion efficiency in the reference group and delta perfusion efficiency in the MB group) was 15% (95% CI, 9%–22%; *P*<0.001). The reduced perfusion efficiency during exercise in the MB group was predominantly driven by the diminution of proximally originating accelerating wave energy during early systole (increase in forward compression wave during exercise was 68% in MB versus 254% in the reference group, *P*<0.001; Figure 4). On the other hand, the reduced perfusion efficiency during exercise in the CMD group was driven by perturbations in the microcirculation-derived wave energies: diminished accelerating wave energy during early diastole (increase in backward expansion wave during exercise was 137% in CMD versus 383% in the reference group, *P*<0.001; Figure 4). Typical coronary pressure and flow waveforms, with corresponding wave intensity analysis profiles during peak exercise, are shown in Figure 5.

### Coronary Endothelial Function

Twenty-four patients in the MB, 24 in the CMD, and 14 in the reference groups underwent assessment of coronary endothelial function. Fifty-four percent of patients with MB, compared with 29% in the reference group, had epicardial endothelial dysfunction (*P*=0.126). By the binary definition, 83% of patients with anMB, compared with 64% in the reference group, had microvascular endothelial dysfunction (*P*=0.183). The prevalence of endothelial dysfunction was similar between the MB and CMD groups. MMI was independently associated with epicardial endothelial function (standardized coefficient, −0.723; *P*=0.047), and the model of MMI and impaired coronary perfusion efficiency during exercise accounted for 44% of epicardial endothelial function in patients with anMB (Table 3). None of the biologically plausible variables was associated with microvascular endothelial function.

**Table 3.**
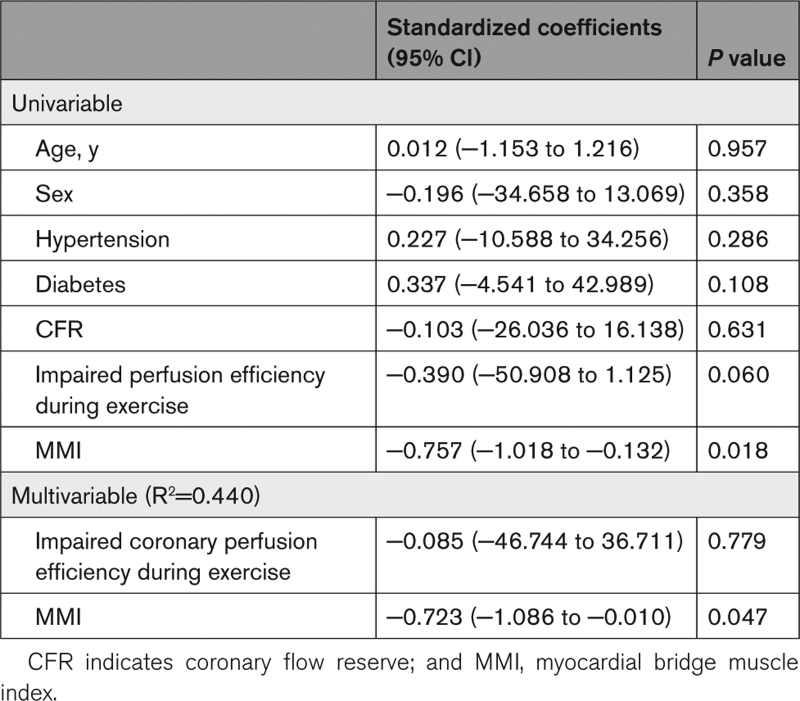
Linear Regression Analysis Testing the Association Between Biologically Plausible Variables and Epicardial Endothelial Function in Patients With Myocardial Bridges

## DISCUSSION

To our knowledge, this is the first study to compare intra-CBF and pressure responses during exercise between patients with MBs, CMD, and normal CFR. Our main findings are the following: (1) patients with a MB have diminished coronary perfusion efficiency during exercise, whereas those in the reference group (no MB and normal CFR) have enhanced perfusion efficiency; (2) the diminished perfusion efficiency in the MB group is driven by reduction of the accelerating cardiac wave energy due to the upstream tunneled segment in early systole; this is in contrast to patients with CMD, where the main driver of perfusion inefficiency is perturbation of the microcirculation-derived wave energies; and (3) patients with a MB have a high prevalence of endothelial dysfunction.

Seminal work from the 1990s first reported the potential of MBs to cause ischemia. The mechanisms by which MBs may reduce myocardial perfusion remain incompletely understood, although several theories have been put forward. The most cited mechanism is delayed decompression of the tunneled segment in early diastole, when most of myocardial perfusion occurs.^3,11^ Tachycardic pacing, in patients with MBs, led to incomplete decompression of the tunneled segment in early diastole, as well as reducing the diastolic perfusion time. Others have since reported that certain MBs can lead to perturbed diastole-specific pressure indices in response to dobutamine in the catheter laboratory,^4,12,13^ which may be associated with inducible ischemia on noninvasive stress imaging.^14^

Over the past few years, we have developed a clinical model of invasive coronary physiology evaluation during exercise,^7,15,16^ which lends itself to unravelling cardiac-coronary coupling in various pathobiological conditions. In health, coronary perfusion efficiency improves with exercise, but we have observed a paradoxical decrease in perfusion efficiency in both patients with myocardial bridging and CMD. However, the maladaptation to exercise appears to arise from distinct mechanisms in these 2 groups. In patients with CMD, the pathophysiology arises from the microcirculation (resulting in a decrease in accelerating wave energy, as well as an increase in decelerating energy), whereas in patients with MB, the detrimental impact on exercise perfusion arises from the tunneled segment on the epicardial vessel (manifest as a reduction in early systolic accelerating energy).

### Coronary Endothelial Dysfunction

Only a few studies have investigated the prevalence of coronary endothelial dysfunction in patients with MBs. In a study by Pargaonkar et al^4^ 85% of patients with a MB had epicardial endothelial dysfunction using the same diagnostic criteria as our study, whereas Sara et al^8^ have reported epicardial endothelial dysfunction in 60% and microvascular endothelial dysfunction in 58% of patients with a MB. Sara et al reported colocalization of epicardial endothelial dysfunction within the tunneled segments, suggesting a pathophysiological link between the 2. Alterations in wall shear stress have been purported to be the mechanism predisposing the tunneled segments to epicardial endothelial dysfunction. Lifelong systolic compression results in high wall shear stress within the tunneled segment and local structural changes of the endothelial cells, leading to reduced nitric oxide production.^17^ MMI was independently associated with epicardial endothelial dysfunction in our study. Forsdahl et al^18^ have previously reported that MMI >31 on CTCA predicts dobutamine diastolic fractional flow reserve <0.76 with good diagnostic accuracy. Dobutamine diastolic fractional flow reserve <0.76, in turn, predicts the presence of inducible ischemia on exercise stress echocardiography.^14^ These findings have significant clinical implications, as an ischemic substrate can potentially be identified on the basis of the characteristics of a MB on CCTA.

In summary, we have demonstrated that patients with myocardial bridging have 3 main mechanisms of ischemia, namely the hemodynamic consequences of the tunneled segment on accelerating wave energy, and endothelium-independent and endothelium-dependent microvascular dysfunction. In our study, 93% of patients in the MB group had an identifiable substrate for ischemia, with a significant overlap between them. Overall, 73% of patients in the MB group had impaired coronary perfusion efficiency during exercise (driven by deleterious mechanical effects of the tunneled segment on accelerating wave energy), 47% had endothelium-independent microvascular dysfunction, and 83% had endothelium-dependent dysfunction (epicardial and microvascular; Figure S3). As these ischemic substrates may represent distinct therapeutic targets, comprehensive coronary physiology assessment should be advocated in patients with ANOCA and myocardial bridging who have limiting symptoms to both confirm and identify the specific substrate for ischemia.

### Clinical Implications

It is now increasingly recognized that not all MBs are benign anatomic variants. We have demonstrated, for the first time that coronary perfusion efficiency is impaired during exercise in patients with MBs. Furthermore, our data suggest that the diminution of coronary perfusion efficiency during exercise is due to the attenuation of accelerating wave energy during early systole arising from the upstream tunneled segment. We have also demonstrated a high prevalence of both epicardial and microvascular endothelial dysfunction in patients with a MB. These insights demonstrate how patients with benign MBs might be distinguished from those with substrates for ischemia. Development of therapies that specifically target the ischemic mechanisms, as identified in our study, may lead to better patient-centric outcomes than empirical management. Finally, dobutamine is currently used in the catheter laboratory to identify MBs with an ischemic substrate. However, whether dobutamine has similar effects on coronary wave energies as exercise is unknown and should be investigated in future studies.

### Study Limitations

This is a small single-center study with some limitations. First, our study participants had a high suspicion of an ischemic substrate based on pretest probabilities, and therefore, our findings should not be extrapolated to MBs that are incidentally discovered or are found in patients with symptoms atypical of angina. Second, all investigations were performed in the LAD artery, potentially limiting the applicability of the findings in other vessels, although MBs mostly involve the LAD artery. Third, we did not assess the bridged vessels with intravascular imaging, which may have uncovered another mechanism of ischemia in the form of proximal vessel atherosclerosis as has been demonstrated in previous studies. Fourth, our study was powered to detect a difference in the primary outcome, change in perfusion efficiency, between the MB and reference groups. Any other comparison is hypothesis generating and should be confirmed in larger studies.

### Conclusions

Patients with ANOCA and a MB exhibit impaired coronary perfusion efficiency during exercise, which is predominantly due to diminution of the accelerating wave energy in early systole; this is secondary to the tunneled segment in the upstream epicardial vessel. This mechanism is distinct to that leading to coronary perfusion inefficiency in patients with CMD. Furthermore, patients with a MB have a high prevalence of epicardial endothelial dysfunction, which correlates with the MMI on CCTA. Comprehensive coronary physiology assessment with wave intensity analysis helps distinguish MBs with an ischemic substrate from benign MBs. Future studies are warranted to assess whether patients with anMB may benefit from distinct pharmacological or mechanical therapies targeting these abnormalities.

## ARTICLE INFORMATION

### Sources of Funding

This work is supported by grants from the UK Medical Research Council (MR/T029390/1), the British Heart Foundation (FS/16/49/32320 and FS/13/15/30026), the UK National Institute for Health Research (through the Biomedical Research center award to Guy’s and St Thomas’ Hospital and King’s College London), and the Foundation Leducq.

### Disclosures

None.

### Supplemental Material

Figures S1–S3

## Supplementary Material


