# Supplementary material for: Characterizing Mechanisms of Ischemia in Patients With Myocardial Bridges
Source: Circ Cardiovasc Interv. 2023 Nov 6;17(1):e013657. doi: 10.1161/CIRCINTERVENTIONS.123.013657 (PMC10782941; doi:10.1161/CIRCINTERVENTIONS.123.013657)
Supplement: Supplementary file 1 [file hcv-17-e013657-s001.pdf]

Supplemental material

Figure S1. Cardiac wave energies during a cardiac cycle.

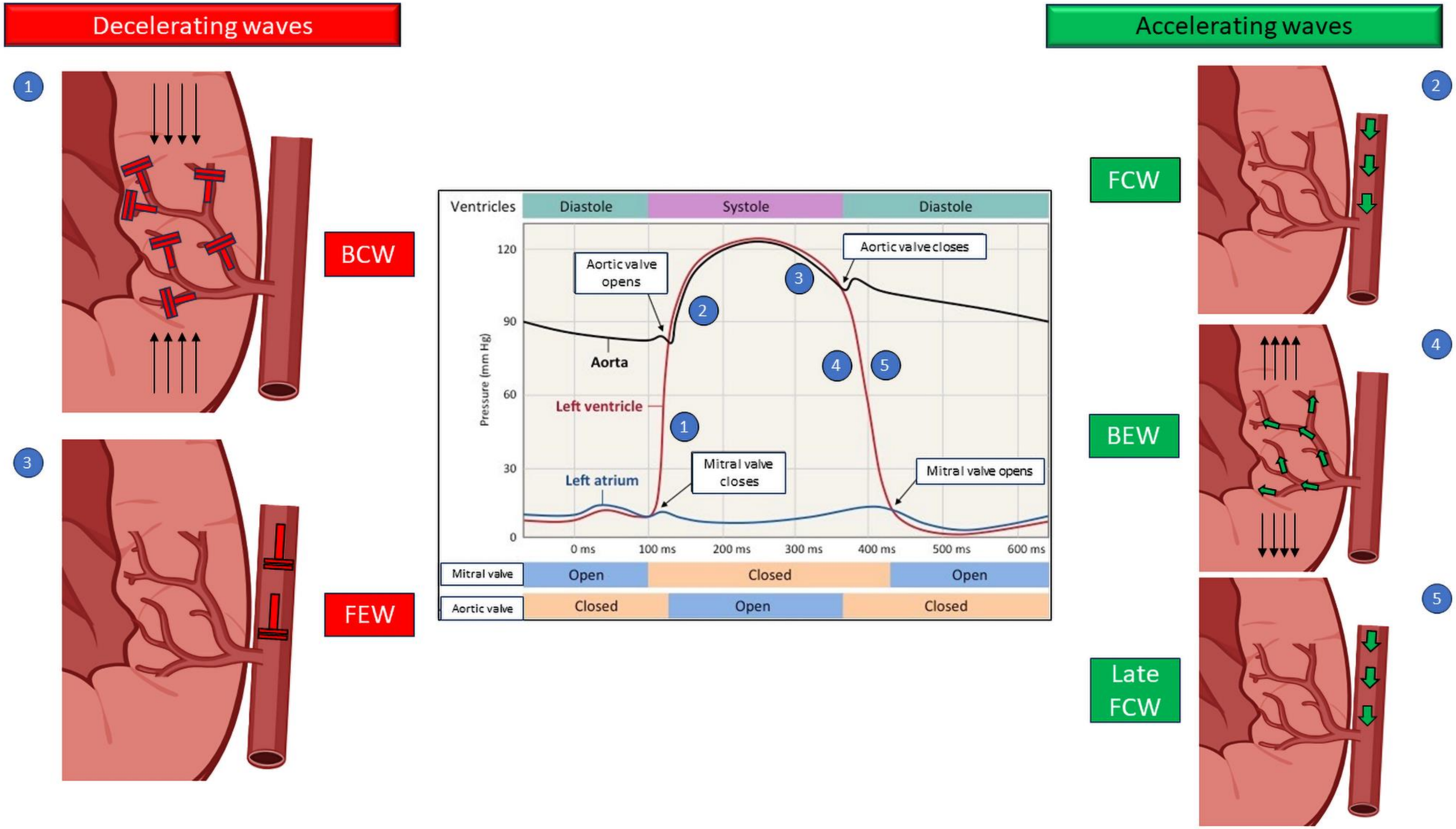

Figure legend

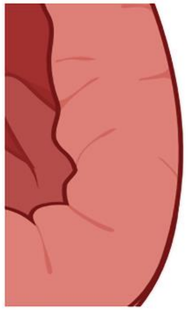

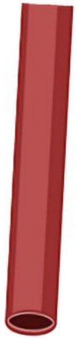

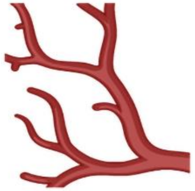

1. Backward compression wave (BCW): causing flow deceleration due to compression of microvasculature during isovolumetric contraction;
2. Forward compression wave (FCW): causing flow acceleration due to increased aortic pressure in early systole;
3. Forward expansion wave (FEW): causing flow deceleration associated with the fall in aortic pressure in late systole;
4. Backward expansion wave (BEW): causing flow acceleration due to decompression of the microvasculature in early diastole;
5. Late forward compression wave (late FCW): causing flow acceleration due to augmentation of the aortic pressure during aortic valve closure in diastole

$$\text{Perfusion efficiency} = \frac{Accelerating\ wave\ energy}{Total\ wave\ energy}$$

Figure S2. Myocardial bridge assessment on CCTA imaging.

Total MB length: 35.2mm; MB depth: 3.3mm; MB depth coverage grade: 3; MMI: 105.6

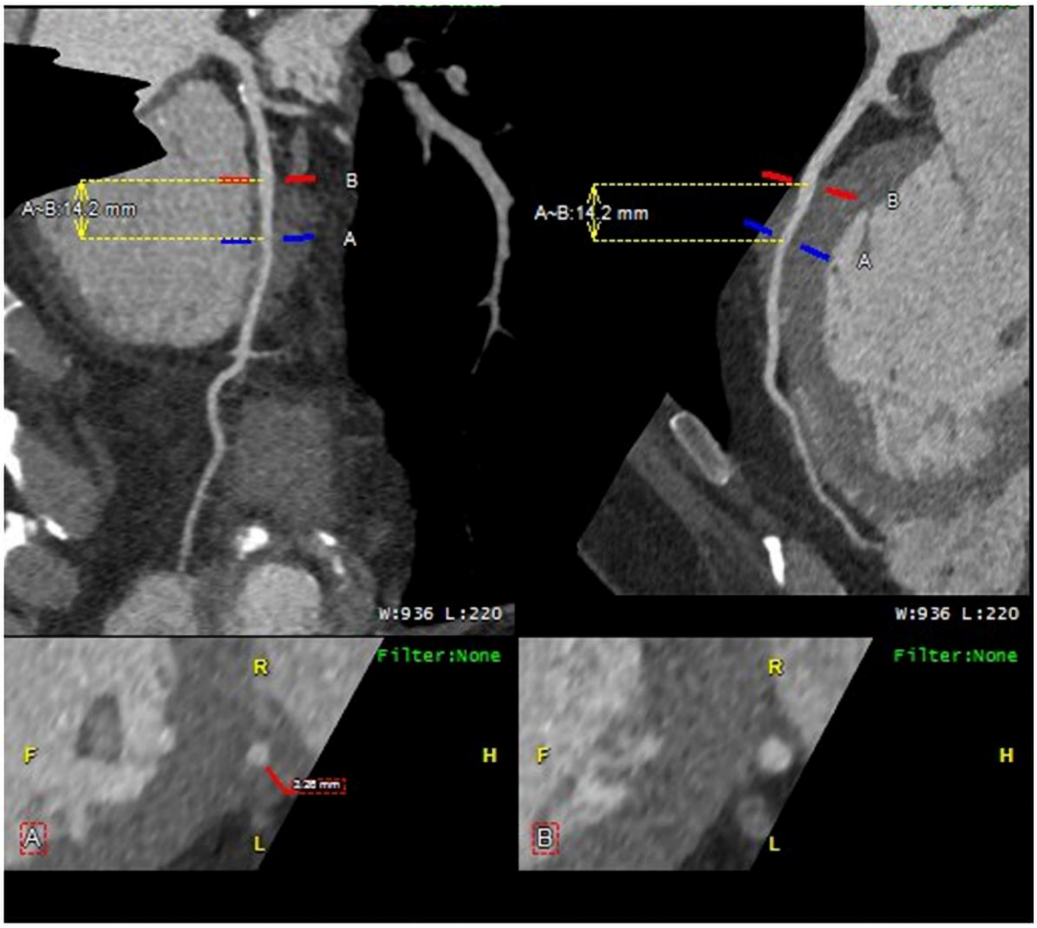

MB: myocardial bridge; MMI: myocardial bridge muscle index

Figure S3. Ischemic substrates in patients with myocardial bridging.

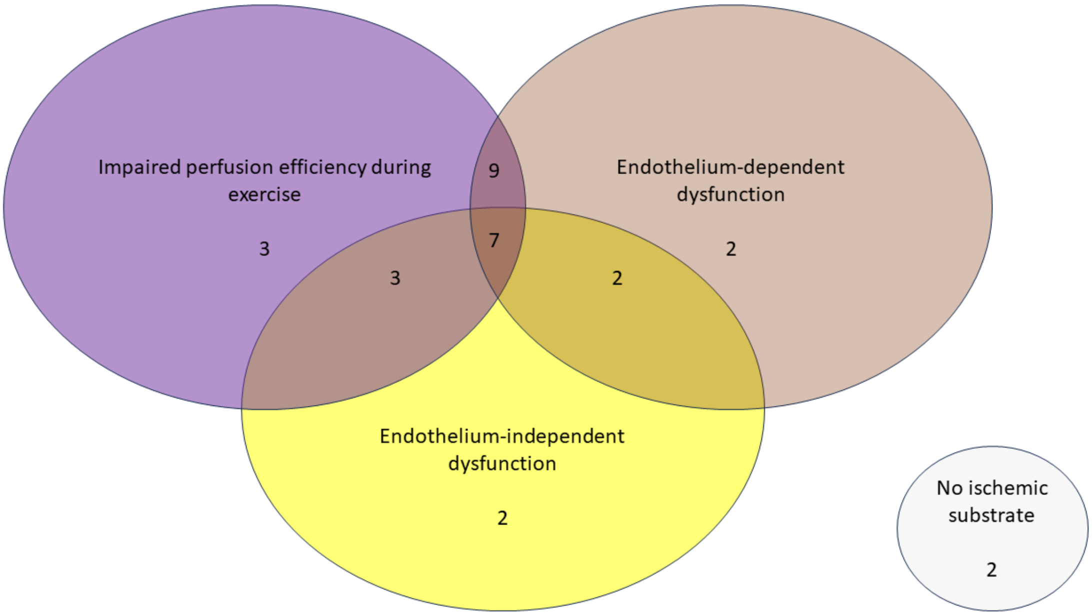

93% of patients with myocardial bridging had an ischemic substrate: 73% of patients had impaired coronary perfusion efficiency during exercise, 83% had endothelial-dependent dysfunction and 47% had endothelium-independent dysfunction.
